# A Lightweight Monocular 3D Face Reconstruction Method Based on Improved 3D Morphing Models

**DOI:** 10.3390/s23156713

**Published:** 2023-07-27

**Authors:** Xingyi You, Yue Wang, Xiaohu Zhao

**Affiliations:** 1 National and Local Joint Engineering Laboratory of Internet Applied Technology on Mines, China University of Mining and Technology, Xuzhou 221008, China; 2 School of Information and Control Engineering, China University of Mining and Technology, Xuzhou 221008, China

**Keywords:** 3DMM, 3D face reconstruction, lightweight network

## Abstract

In the past few years, 3D Morphing Model (3DMM)-based methods have achieved remarkable results in single-image 3D face reconstruction. However, high-fidelity 3D face texture generation has been successfully achieved with this method, which mostly uses the power of deep convolutional neural networks during the parameter fitting process, which leads to an increase in the number of network layers and computational burden of the network model and reduces the computational speed. Currently, existing methods increase computational speed by using lightweight networks for parameter fitting, but at the expense of reconstruction accuracy. In order to solve the above problems, we improved the 3D deformation model and proposed an efficient and lightweight network model: Mobile-FaceRNet. First, we combine depthwise separable convolution and multi-scale representation methods to fit the parameters of a 3D deformable model (3DMM); then, we introduce a residual attention module during network training to enhance the network’s attention to important features, guaranteeing high-fidelity facial texture reconstruction quality; and, finally, a new perceptual loss function is designed to better address smoothness and image similarity for the smoothing constraints. Experimental results show that the method proposed in this paper can not only achieve high-precision reconstruction under the premise of lightweight, but it is also more robust to influences such as attitude and occlusion.

## 1. Introduction

Reconstructing high-fidelity 3D human faces is a long-standing problem in the multimedia and computer vision communities. Faithfully reconstructing 3D faces is a crucial prerequisite for many downstream applications, including face editing [1], virtual avatar generation [2,3], face alignment [4], and recognition [5]. The proposed process aims to estimate a realistic 3D facial representation that predicts face geometry, appearance, expression, and scene lighting from the input source.

Methods of traditional 3D facial reconstruction are multi-eye stereo vision matching [6,7], 3D morphing models (3DMM) [8,9], and shape from shading [10]. However, most of these methods require high-fidelity 3D face data to build the 3D face models [11], which can be problematic. In addition, general high-fidelity 3D data scans are difficult to set up [12]. Therefore, there are several constraints that limit the wide application of 3DMM [13] For more than a decade, most existing models have used no more than 300 training scans. However, this small training set is inadequate to describe the full variability of human faces [14,15].

Human facial images are mostly composed of non-linear data, such as expressions and wrinkles [16,17], and the reconstruction of texture details based on linear 3DMMs has been unsatisfactory [18]. Recently, many attempts have been undertaken to address the lack of detail in 3DMMs by adding non-linearity to the parametric model. For example, a linear 3DMM was replaced with a completely non-linear 3DMM [16,17,19]. In other research, non-linearity was added as a complement to the 3DMM coarse reconstruction [20,21,22,23]. In these methods, facial details were either represented in geometry using a displacement map or encoded into the appearance using a detailed texture (or albedo) map.

With the development of deep convolutional neural networks (CNNs), an increasing number of experts and scholars have begun to use weakly supervised methods of deep CNNs to apply a 3DMM coefficient regression [24]. However, the network structure used by these methods is complicated, and the model’s operational efficiency is low. At the same time, the inference time is long and the model parameter space is large, so they are unsuitable for certain applications.

In order to solve this issue and create an efficient model, this paper proposes a novel and efficient network structure design called Mobile-FaceRNet. The proposed model reduces the computational complexity and loss of network performance while achieving the expected effect because it uses a lightweight network to replace the traditional deep CNN for a 3DMM regression coefficient. In addition, a multiscale feature extraction fusion and residual attention models were added to the lightweight network model training to restore more refined facial details by observing the key areas that reflect the facial details. Simultaneously, a new loss function was also designed to constrain the smoothness of the learned 3D face model and better establish the similarities and differences between the input face image and the rendered image. This enables the proposed method to achieve higher accuracy in a more lightweight manner. This article contributes as follows:

- An end-to-end lightweight neural network (Mobile-FaceRNet) is created, an encoding–decoding framework is designed, and the existing 3DMM is improved to effectively and quickly reconstruct a more accurate 3D face model.
- A residual attention model and a multiscale feature extraction fusion model are added to quickly obtain global information while prioritizing. Subsequently, a higher focus is laid on some of the key information by superimposing the weight values of different regions of interest.
- A new loss function is designed that smoothly constrains the learned 3D face model. Simultaneously, intensive training is conducted on the feature points with the loss function, which obtains larger loss values than those obtained during the previous training.
- A comparison of the methods using the challenging AFLW2000-3D and AFLW-LFPA datasets demonstrates that the proposed method achieves significantly improved performance on 3D face reconstruction and face alignment tasks.

The rest of this paper is organized as follows. The related studies on 3D face reconstruction are reviewed in Section 2. Section 3 provides a detailed description of the proposed method. Section 4 presents the experiment setup and a discussion of the results. Finally, some concluding remarks are presented in Section 5.

## 2. Related Work

### 2.1. 3DMM

Blanz and Vetter proposed the first 3DMM model that provided an improved basis for subsequent 3DMM models [8]. Paysan et al. created the Basel face model (BFM) to fill in the gaps in the 3D face reconstruction dataset [25]. In addition, Amberg et al. used non-rigid registration, and their method of dividing face attributes provided new possibilities for 3D face reconstruction in terms of image registration and multilinear improvements [21]. However, they did not eliminate the linear templates. Later, Bolkart and Wuhrer demonstrated how to use joint optimization of the model parameters and group registration of 3D scans to directly estimate a multilinear model from 3D scans, and then further developed their approach into a non-linear 3DMM model [26].

### 2.2. Face Alignment

Face alignment is an important aspect of 3D face reconstruction. The earlier 2D face alignment methods, such as cascaded pose regression developed by Dollar et al., mainly located a set of sparse face key points [27]. Its main operation was a vector addition, which could still be attributed to the regression problem. With the development of deep learning, some scholars have gradually applied CNN methods to 2D face alignment, despite certain inherent limitations [28]. For example, 2D face alignment can only detect feature points that are visible in a 2D plane. When the pose of the face is large or occlusions occur, 2D face alignment cannot detect all the feature points within the range of the face. Subsequently, researchers began to study 3D face alignment methods [29]. Tulyakov et al. designed a cascaded regression framework to match real 3D face feature points and solved the problem of invisible feature points caused by self-occlusions, making important contributions to the preservation of face shape and evaluation of the orientation of the face [30]. However, a cascaded regression is still needed.

### 2.3. 3D Face Reconstruction

Roth et al. proposed a method for reconstructing a 3D face model using albedo information that reconstructed faces from a low-quality dataset with fewer images [31]. Dou et al. adopted an end-to-end training network to avoid complex 3D rendering, discarding the original methods of initializing RGB images and 3D facial expression rendering [32]. Additionally, they proposed a method for adding various details after the geometric model had been built [33]. However, these methods were subject to the limitations of the 3DMM model implementation framework and, therefore, could not handle fine changes outside the subspace, such as hair or details in the lips or eyes.

A volumetric CNN was proposed to directly map the image pixels to a full 3D facial structure without being restricted in the model space, but it required a complex network and lengthy processing time to predict the voxel data [22]. More recently, Feng et al. took a different approach by storing the 3D facial geometry into a UV position map and training an encoder–decoder CNN to directly regress the complete 3D facial structure along with the semantic information from a single image [34]. Subsequently, Deng et al. used a weakly supervised method to regress the 3DMM parameters, which achieved state-of-the-art performance in faces with large poses and unbalanced illuminations [35]. Tu et al. developed a new self-critic learning-based approach that could effectively improve the 3D face model learning procedure and produce a better model [36]. However, this method still required 2D face feature point information as support.

Therefore, compared with these works, this study proposed training a higher- performance network—Mobile-FaceRNet—and designed a multiscale feature preprocessing module to provide richer multiscale feature information for the subsequent prediction network. Simultaneously, the encoding–decoding structure of the prediction sub-network is reasonably designed. The image feature information is different according to the various prediction components of each network. Furthermore, a residual attention mechanism module is introduced to effectively improve the speed and ability of network feature extraction. These combined improvements greatly enhance the accuracy of the 3D face reconstruction and dense alignment.

## 3. Methods

The framework and details of the proposed method for simultaneous 3D face reconstruction and 3D face alignment that fits a 3DMM with an efficient CNN are discussed in this section.

**Figure 1:**
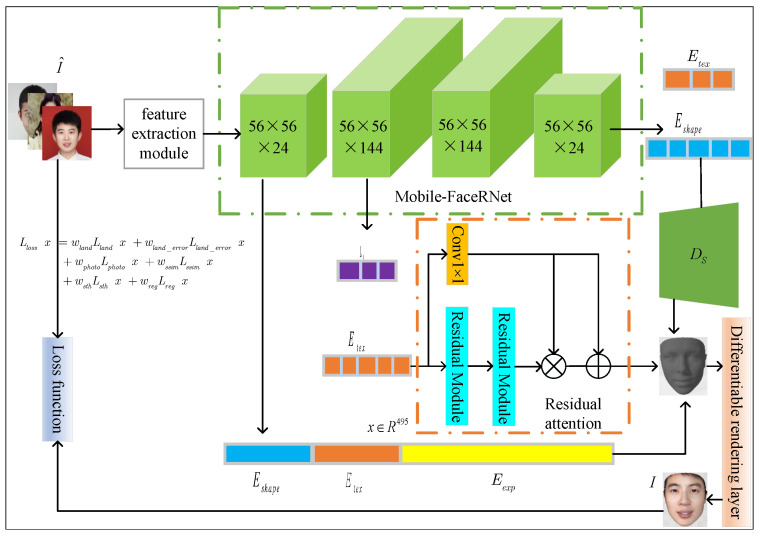
Pipeline overview of the proposed Mobile-FaceRNet.

The overall structure of the network is shown in Figure 1, which consists of a feature extraction module, an encoder–decoder module, and a loss function. The feature extraction module, indicated by the blue border in Figure 1, obtains fused features with richer information by densely connecting each feature extraction unit to input into the encoder–decoder module. The specific feature extraction module introduction will be shown in Section 3.2. The encoder–decoder module is represented by the green and orange parts in Figure 1. The residual attention mechanism is introduced on the basis of the improved Mobilenetv2; it encodes and decodes the extracted features and obtains the 3DMM coefficients, camera parameters, and spherical harmonic illumination coefficients of the face. Subsequently, two fully connected layers are passed to the 3D face model, improving the shape and texture. The corresponding 3D face model is reconstructed by adding spherical harmonic illumination. The specific parameter introduction and the Mobile-FaceRNet network structure will be shown in Section 3.1 and Section 3.3. The overall network is trained by backpropagation. The loss function part in Figure 1 is a new perceptual loss function designed by us to better address smoothness and image similarity for the smoothing constraints. The specific introduction will be shown in Section 3.4.

### 3.1. Parameters

#### 3.1.1. DMM Parameters

The proposed method uses a parameterized 3D face geometry model as the initial face geometry model, which is expressed as $S=\left\{s_{i}\in R^{3}|1\leq i\leq N\right\}$, where N=35,709 is the number of vertices. At the same time, the parameterized face texture model is expressed as $T=\left\{t_{i}\in R^{3}|1\leq i\leq N\right\}$. These models are used for the initial face texture models, which are expressed as

(1) $$S=S\left(\alpha ,\delta \right)=\bar{S}+E_{shape}\alpha +E_{\exp}\delta$$

(2) $$T=T\left(\beta \right)=\bar{T}+E_{tex}\beta$$

where $\bar{S}$ is the average face geometry model and $\bar{T}$ is the average face texture model. $E_{shape}\in R^{3N\times 199}$, $E_{tex}\in R^{3N\times 199}$, and $E_{\exp}\in R^{3N\times 64}$ are the principal component analysis (PCA) bases for face shape, texture, and expression, respectively. $\alpha \in R^{199}$, $\beta \in R^{199}$, and $\delta \in R^{64}$ are the shape, texture, and expression coefficients corresponding to the 3D face model, respectively. $\bar{S}$, $\bar{T}$, $E_{shape}$, and $E_{tex}$ from the Basel Face Model 2009 database [8], and $E_{\exp}$ is from the FaceWarehouse database [37].

The existing PCA bases for face shape and texture were improved by building two fully-connected layers, $FC_{shape}$ and $FC_{texture}$, respectively.

The fully-connected layer $FC_{shape}=199\times 35,709\times 3$, where the input is 199, output is 107,127, and PCA-based $E_{S}$ of the face shape in the 3DMM has an initial weight to obtain an improved face shape $\bar{S}$. Similarly, the size of the fully-connected layer $FC_{texture}=199\times 107,127$, where $E_{T}$ is the initial weight, and the improved face shape $\bar{S}$ and texture $\bar{S}$ models are obtained. The final 3D face reconstruction calculations are expressed as:

(3) $$S=S\left(\alpha ,\beta \right)=\bar{S}+S_{new\_shape}+E_{\exp}\delta$$

(4) $$T=T\left(\beta \right)=\bar{T}+T_{new\_texture}$$

#### 3.1.2. Camera Parameters

A camera model was used to transform the face mesh model from a 3D space to a 2D plane. Similar to past research [38], a full perspective projection model was used. The position and orientation of the camera in the world coordinate system are represented by the rotation matrix $R\in SO\left(3\right)$ and translation vector $m\in R^{3}$, respectively, and are expressed as:

(5) $$q=\Pi \left(Rp+m\right)$$

#### 3.1.3. Spherical Harmonic Illumination Coefficient

It was assumed that the illumination was low-frequency and approximated the face surface as a Lambert surface. Based on these two assumptions, spherical harmonics were used to represent illumination [39]. The vertex color $C\left(t_{i},n_{i},\gamma \right)$ was calculated from the mesh vertex texture $t_{i}\in R^{3}$, mesh vertex normal vector $n_{i}\in R^{3}$, and illumination coefficient $\gamma \in R^{27}$, expressed as

(6) $$C\left(t_{i},n_{i},\gamma \right)=t_{i}\cdot \sum_{b=1}^{B^{2}}r_{b}H_{b}\left(n_{i}\right)$$

where $\gamma =\left\{r_{b}\in R^{3}|1\leq b\leq B^{2}\right\}$ is the corresponding illumination coefficient. $H_{b}:R^{3}\to R$ is the spherical harmonic basis function and the first three orders (b = 3) were used.

### 3.2. Feature Extraction Module

DenseNet proposes a dense connection mode (dense connectivity) that connects each layer with subsequent layers to output feature maps of the same size [40]. This dense connection ensures that information flows between the layers, resulting in a more effective transfer of features and gradients in the network. Additionally, the dense connection improves the gradient disappearance problem caused by deepening the CNNs. At the same time, the dense connection enables the final feature map that is output from the network to synthesize the features of all the levels. Features at different levels in deep CNNs represent different information. In a lower stage, the receptive field of the network is smaller, more attention is paid to the details of the image, and the semantics are less clear. At a higher stage of the network, the feature receptive field becomes larger and the semantic features are more accurate, but the ability to represent the details becomes weaker. The fusion of different levels of features can make full use of the semantic information of high-level features and the detailed information of low-level features, which improves the accuracy of 3D face reconstruction and dense alignment.

**Figure 2:**
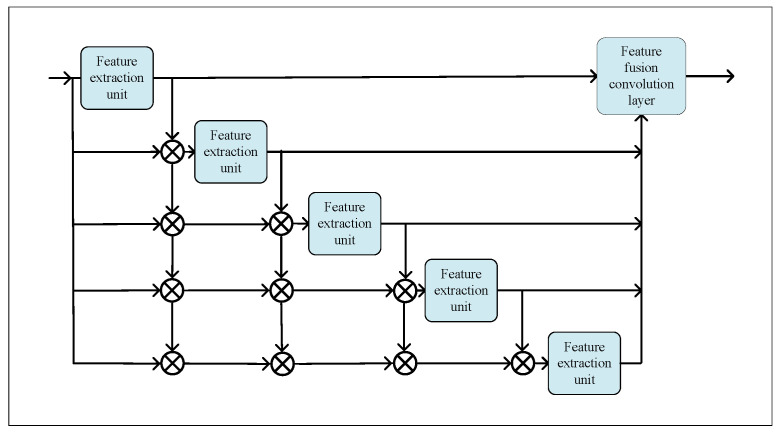
Structural diagram of the feature extraction module.

The proposed feature extraction module that obtains adequate rich information to feed into the encoder–decoder module is shown in Figure 2. First, the image was preprocessed using two convolutional layers with a kernel size of 3 × 3 and a channel number of 8. Second, the outputs of each feature extraction unit were fused by a dense connection to obtain multiscale fusion features. However, simply forming a feature extraction unit through ordinary convolutional layers requires greatly deepening the network to achieve a sufficiently large receptive field, which significantly increases the number of network parameters.

To solve this problem, the proposed feature extraction unit was designed to consist of a convolutional layer with a kernel size of 1 × 1 and 3 ResNet modules [41] with a kernel size of 3 × 3. The number of output channels was 8. The 3 × 3 convolutional layers of the middle module used atrous convolution, which was different from the first and third ResNet modules. In addition, the dilation rate was set to 3 instead of the default 1. Using a convolutional layer with a kernel size of 1 × 1 reduced the dimension of the feature map and the number of network parameters, and improved computational efficiency. Applying atrous convolution enable the convolutional layer to expand the receptive field of output features while keeping the parameters unchanged.

The size of the equivalent receptive field *R* for an atrous convolutional layer with an expansion rate *d* and kernel size *K* is expressed as:

(7) R=(d−1)×(K−1)+K

This study adopted a 3 × 3 convolutional layer with a dilation rate of d=3, corresponding to a receptive field size of 7. Stacking convolutional layers resulted in a larger receptive field. By stacking two convolutional layers with kernel sizes of $K_{1}$ and $K_{2}$, the final equivalent receptive field size is expressed as:

(8) $$K=K_{1}+K_{2}-1$$

According to Equations (7) and (8), the receptive field size of the output feature map for the designed feature extraction unit was 11, and the receptive field of the output feature map for the densely connected multiscale feature fusion module was 51. When atrous convolution was not used, these values were 7 and 31, respectively. Obviously, using the designed network structure ensured that the features of a larger receptive field could be obtained in the case of a shallower network depth and a smaller number of parameters. At the same time, this made the output feature map receptive field of each feature extraction unit more different, allowing the network to obtain more informative fusion features to input into the encoder.

### 3.3. Network Structure

A novel and efficient network structure named Mobile-FaceRNet was designed, which was based on Mobilenetv2 [42] and transferred the input RGB image into parameters. This model applied a depthwise separable convolution, multiscale representation, and residual attention mechanism for 3D face alignment and 3D face reconstruction tasks for the first time. The components of the Mobile-FaceRNet architecture are listed in Table 1.

The proposed encoder–decoder module was based on Mobilenetv2 [42]. The encoder part obtained higher-level coding information through continuous downsampling and convolution. After the decoder part continuously upsampled, it fused with the shallow coding information through skip connections. Channel connection was used to fuse different coding information, which was different from directly adding high- and low-level coding information [43]. The direct summation method ignores the differences between the different levels of coding information, and the channel connection method can completely retain different coding information. This study introduced a residual attention mechanism in the decoder to highlight the focused parts of the task more effectively [44].

The residual attention module was divided into two branches: trunk and soft mask. In contrast to spatial or channel attention mechanisms, a residual attention mechanism generates weight information for all the elements of the feature map. Its purpose is to inform the network which coding information needs more attention. The output H of the residual attention module is expressed as

(9) $$H_{n,c}=\left(1+G_{n,c}\left(x\right)\right)\times F_{n,c}\left(x\right)$$

where *n* represents the value at all the spatial positions. $c\in \left\{1,2,\cdots ,C\right\}$ is the index of the channel when the residual attention module is given input *x*. F(x) and M(x) are the outputs of the main and soft mask branches, respectively.

**Figure 3:**
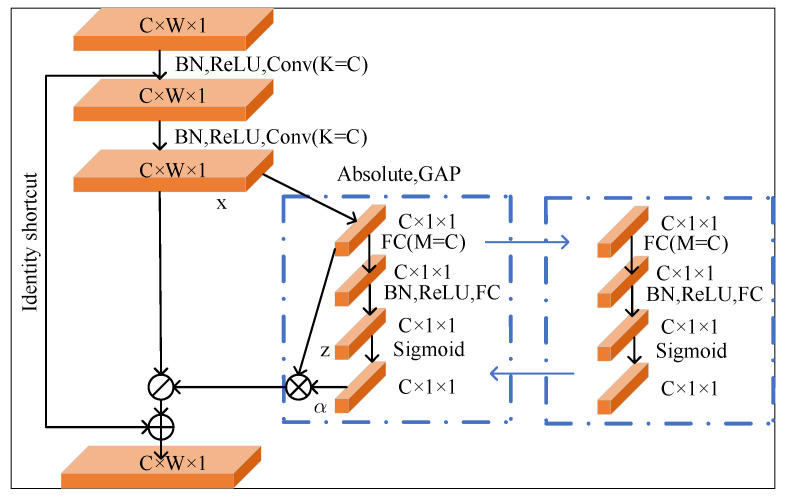
Residual attention module.

The structural design of the proposed residual attention module is shown in Figure 3. The main branch of the residual attention module is a convolutional layer; the size of the convolution kernel is 1 × 1, and the number of channels is half the number of input feature maps. The 1 × 1 convolution of the backbone channel can effectively reduce the number of feature channels, reduce the computational complexity, and merge the features of each channel simultaneously. The soft mask branch is composed of two residual modules to generate attention information, which act as feature selectors to enhance the good features and suppress noise from the backbone features. The residual attention module adopts the concept of residual learning, which can save the output characteristics of the main branch and avoid weakening of the deep feature map caused by the stacking of multiple attention modules.

The improved lightweight network was used to obtain the 3D face parameter $x\in R^{495}$ that needs to be regressed, including a 3DMM shape parameter $\alpha \in R^{199}$, 3DMM texture parameter $\beta \in R^{199}$, 3DMM expression parameter $\delta \in R^{64}$, and camera rotation $R\in SO\left(3\right)$. The camera translation $m\in R^{3}$ and spherical harmonic illumination parameter $\gamma \in R^{27}$ are expressed as *m* and *g*, respectively, in:

(10) $$x=\left(\alpha ,\beta ,\delta ,R,m,\gamma \right)$$

### 3.4. Loss Function

The loss function is the key to ensuring the smooth progress of the entire end-to-end network and is an important part of obtaining a realistic 3D face reconstruction model. The proposed loss function is expressed as

(11) $$\begin{array}{c} L_{loss}\left(x\right)=\omega_{land}L_{land}\left(x\right)+\omega_{land\_error}L_{land\_error}\left(x\right)\\ +\omega_{photo}L_{photo}\left(x\right)+\omega_{ssim}L_{ssim}\left(x\right)+\omega_{sth}L_{sth}\left(x\right)+\omega_{reg}L_{reg}\left(x\right) \end{array}$$

where $L_{land}\left(x\right)$ and $L_{land\_error}\left(x\right)$ are the loss functions of the feature point alignment and enhancement training, respectively. $L_{photo}\left(x\right)$ is the loss function of the difference between the original image and the 3D face rendering image. $L_{ssim}\left(x\right)$ is the difference between the original image and the 3D face rendering image. The loss function of the structural similarity index measure (SSIM), $L_{smooth}\left(x\right)$ is the 3D face model smoothness constraint loss function and $L_{reg}\left(x\right)$ is the regularization term loss function. The weights were set as $\omega_{land}=400$, $\omega_{land\_error}=2000$, $\omega_{photo}=100$, $\omega_{ssim}=2$, $\omega_{smooth}=50$, and $\omega_{reg}=1$ to balance the loss function of each part.

Feature point loss function: This method uses the feature points of 2D face images as weakly supervised information to train the neural network. At the same time, the relatively advanced facial feature point detection algorithm is used to detect the 68 key points of the face image in the training set [45]. The loss function $L_{land}\left(x\right)$ is expressed as

(12) $$L_{land}\left(x\right)=\sum_{i=1}^{68}\omega_{i}\times {∥\nu_{k_{i}}-\nu_{i}^{{}^{\prime }}∥}_{2}^{2}$$

where $\omega_{i}$ is the weight corresponding to the feature point. The weight of the 52 feature points fixed in the middle of a face is 1, and the weight of the 16 contour feature points in the boundary position is 0.5. $\nu_{i}^{{}^{\prime }}\in R^{2}$ is the real label of the 2D feature point of the face, $k_{i}\in \left\{1,2,\ldots ,N\right\}$ is the vertex index of the corresponding 3D face model, and $\nu_{k_{i}}$ is the coordinate of the reconstructed 3D face model projected to the pixel plane.

A loss function $L_{land\_error}\left(x\right)$ was added after the fifth iteration to strengthen the training of feature points with relatively large errors, which is expressed as

(13) $$L_{land\_error}\left(x\right)=\sum_{i=1}^{52}e_{i}\times {∥\nu_{k_{i}}-\nu_{i}^{{}^{\prime }}∥}_{2}^{2}$$

where $e_{i}$ is the average error of the 52 fixed feature points in the training of the previous iteration.

Pixel loss function: The goal of the pixel loss function $L_{photo}\left(x\right)$ is to make the rendered and input images as close as possible, render the reconstructed 3D face model to the pixel space, and align it with the input monocular face image. The proposed method used a differentiable renderer to render the 3D face model to the 2D plane [8]. The rendered image was matched with the input monocular face image, and their similarity in pixel space was compared. The loss function $L_{photo}\left(x\right)$ is expressed as

(14) $$L_{photo}\left(x\right)=\frac{1}{n}\sum_{i\in V}^{\;}{∥I_{i}-I_{i}^{{}^{\prime }}∥}_{2}$$

where *V* is the set of all the projected face area pixels on the pixel plane, and *n* is the number of pixels in *V*. The rendering of the 3D face model is the color of the input monocular face image at position i and the resulting image at position i after inputting the face area pixels.

SSIM loss function: The goal of the SSIM loss function is to guarantee the structural similarity of the input and rendered images. The texture of the 3D face model can be better reconstructed by adding the SSIM loss function, which is expressed as

(15) $$L_{ssim}\left(x\right)=1-\frac{\left(2\mu_{I}\mu_{I^{{}^{\prime }}}+c_{1}\right)\left(2\sigma_{II^{{}^{\prime }}}+c_{2}\right)}{\left(\mu_{I}^{2}+\mu_{I^{{}^{\prime }}}^{2}+c_{1}\right)\left(\sigma_{I}^{2}+\sigma_{I^{{}^{\prime }}}^{2}+c_{2}\right)}$$

where $\mu_{I}=\frac{1}{n}\cdot \sum_{i\in V}^{\;}I_{i}$ is the set of all the projected face area pixels on the pixel plane, *n* is the number of pixels in *V*, and $\sigma_{II^{{}^{\prime }}}$ is the covariance of the visible area texture of the input and rendered images. and $\sigma_{I^{{}^{\prime }}}^{2}$ are the variances $\sigma_{I^{{}^{\prime }}}^{2}$ of the visible area texture in the input and rendered images, respectively. Here, $c_{1}=0.01^{2}$ and $c_{2}=0.03^{2}$. In addition, $\mu_{I^{{}^{\prime }}}=\frac{1}{n}\cdot \sum_{i\in V}^{\;}I_{i}^{{}^{\prime }}$, and are the average textures of the visible area in the input and rendered images, respectively.

Smoothness Constraint Loss: A smoothness constraint loss of a 3D face ensures the smooth shape of a reconstructed 3D face model and prevents the 3D face reconstruction results from turning over and having a rough surface. The smoothness constraint is expressed as

(16) $$L_{sth}=\frac{1}{N}\sum_{i=1}^{N}{∥\frac{1}{d_{i}}\sum_{j\in Adj_{i}}\left(v_{i}-v_{j}\right)∥}_{2}^{2}$$

where *N* is the number of vertices of the 3D face model, $d_{i}$ is the degree of the i-th vertex of the 3D face model, $Adj_{i}$ is the set of neighbor indexes of the i-th vertex of the 3D face model, and $v_{i}$ is the i-th vertex of the 3D face model coordinates.

Regularization: Regularization items were added to reasonably constrain the network and ensure the final integrity of the 3D reconstruction, which is expressed as:

(17) $${L_{r}}_{eg}=\sum_{i}\omega_{\alpha }{∥\alpha ∥}^{2}+\omega_{\beta }{∥\beta ∥}^{2}+\omega_{\delta }{∥\delta ∥}^{2}$$

Here, $\omega_{\alpha }=2\times 10^{-5}$, $\omega_{\beta }=2\times 10^{-2}$, and $\omega_{\delta }=4\times 10^{-4}$.

## 4. Experiments

### 4.1. Implementation Details and Datasets

The BFM was used as the 3D deformable face model. The face image dataset used the CelebA [46] and 300W-LP [47] attribute datasets, and the images were balanced and optimized in advance. A total of approximately 120,000 clear facial images with a relatively uniform distribution were obtained for the 3DMM training. Data enhancements were made to these images, including flipping (horizontal flipping), random rotation (rotating from $-30^{\circ }$ to $-30^{\circ }$ clockwise based on the center point), and simulated lighting. During the training process, the image was either flipped or rotated at random, with a probability of 50% for each application. The simulated lighting operation randomly multiplied the RGB color channel of the face image by 0.6 to 1.2, and the three channels were operated independently. The probability distribution was a uniform distribution of 0.6 to 1.2.

The test dataset used the AFLW2000-3D and AFLW-LFPA facial image datasets. AFLW2000-3D is composed of the first 2000 images in the AFLW database and their 3D information [47]. The 3D information was obtained through a 3DMM reconstruction and contained 68 feature points. AFLW-LFPA is another extension of the AFLW dataset [28]. It contains face images with multiple poses and views, a balanced yaw angle distribution, and 34 face key points.

Using the network model described above for training, the size of the input images was set to 64 × 64 × 3 pixels, and the number of vertices was 35,709, which is the same as in Ref. [28]. An Adam optimizer was used to optimize the model with a learning rate of 0.001 and a batch size of 4. The proposed network was trained on a Lenovo P720 graphics workstation.

### 4.2. Comparative Experiment

#### 4.2.1. 3D Face Alignment

**Figure 4:**
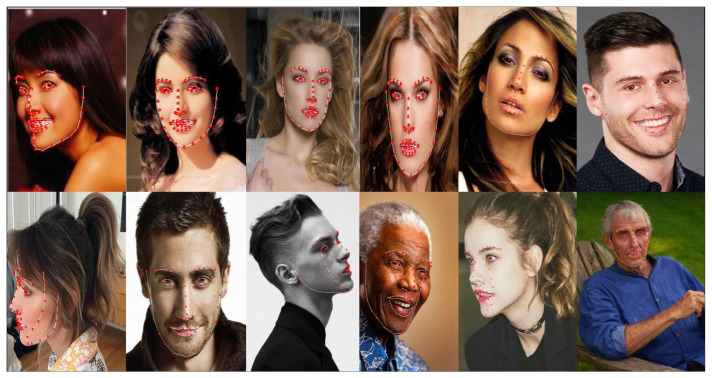
Dense face alignment effect.

Face images were randomly selected for qualitative testing from the ALFW2000-3D dataset, as shown in Figure 4. The normalized mean error (NME) was used as an index to evaluate the performance of the algorithm [47]. The normalized average error was normalized according to the size of the face-bounding box, which is expressed as

(18) $$NME=\frac{1}{T}\sum_{k=1}^{N}\frac{{∥m_{k}-n_{k}∥}_{2}}{d}$$

where *T* is the number of vertices and d is the square root of the product of the length and width of the real bounding box of the face, which is calculated as $d=\sqrt{\omega_{bbox}\times h_{bbox}}$. In addition, $m_{k}\in R^{2}$ and $n_{k}\in R^{2}$ are the predicted point coordinates and label on the test set, respectively.

The absolute value of the yaw angle was divided into three types: I $\left(0^{\circ },30^{\circ }\right]$, II $\left(30^{\circ },60^{\circ }\right]$, and III $\left(60^{\circ },90^{\circ }\right]$. A total of 574 sheets were randomly selected for testing; thus, the ratio of the face image at each angle was 1 to ensure that the result was evenly distributed. A total of 68 sparse feature points were used to measure the face alignment effect [48]. The results of the proposed method compared to those of the other methods on the AFLW2000-3D (68 feature points) [27] and AFLW-LFPA (34 feature points) datasets are listed in Table 2. The evaluation standard used the normalized average error (%). As the data decreased in number, the alignment effect improved. Here, “-” indicates that there is no corresponding data. The data information for the other methods was based on related papers published as the main source. Good robustness and higher accuracy were achieved at different angles of the face poses.

#### 4.2.2. 3D Face Reconstruction

**Figure 5:**
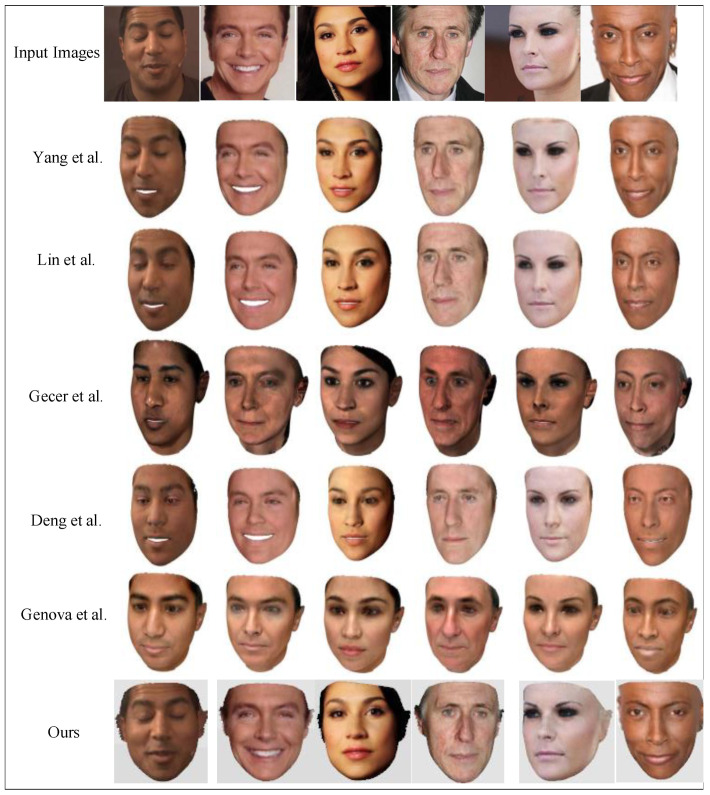
Comparison of qualitative results with other methods (Yang et al. [18], Lin et al. [23], Gecer et al. [19], Deng et al. [35], Genova et al. [38]).

The proposed approach was qualitatively compared against recent learning-based texture reconstruction methods from Refs. [18,19,23,35,38], as shown in Figure 5. The proposed method was superior to the other approaches with high texture reconstruction. From the thickness and shape of the eyebrows to the wrinkles around the mouth and forehead, the proposed texture and shape reconstructions achieved strong identification characteristics in the corresponding input images.

For quantitative comparison, the experiments evaluated the shape reconstruction performance of the proposed method on the CelebA dataset [46]. Additionally, the focus was mainly laid on the criteria for measuring the image-level difference. First, the L1-distance loss was applied as the basic pixel-level criterion. Second, two commonly used image similarity criteria were utilized to evaluate the similarities between the rendered and original input face images, namely the SSIM and peak signal-to-noise ratio (PSNR). With regard to the human face problem, the PSNR is expressed as

(19) $$\mathrm{PSNR}\left(I,\bar{I}\right)=10\cdot \log_{10}\left(\frac{255^{2}}{\mathrm{MSE}\left(I,\bar{I}\right)}\right)$$

where *I* and $\bar{I}$ are the 2D image of the original face and the projected 2D image of the reconstructed face, respectively.

**Figure 6:**
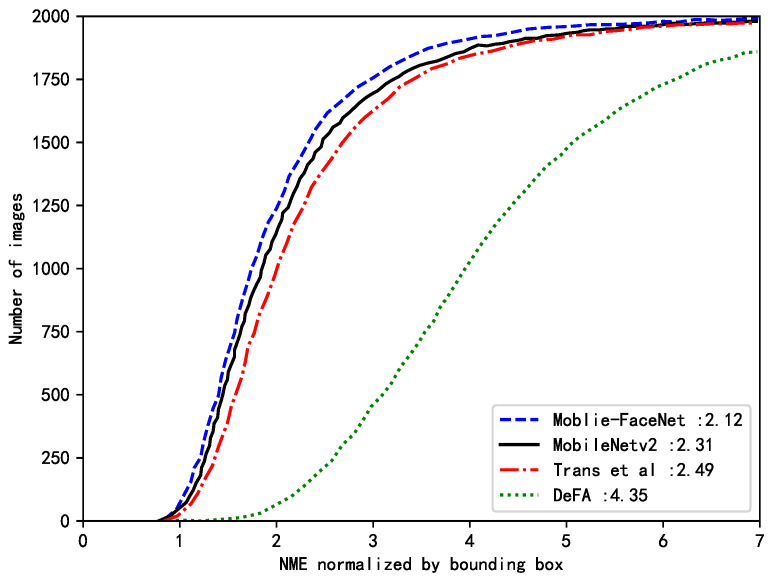
3D reconstruction performance (CED curves) on the AFLW2000-3D dataset (Trans et al. [17]).

Two well-known pretrained face recognition networks were also leveraged to map from the image space to the feature space and evaluate the difference between the rendered and input face images in the facial feature space. The two facial recognition networks that were adopted were LightCNN and evoLVe [55] because of their state-of-the-art performances and wide acceptance [23]. In summary, the difference was calculated between two face images at the pixel level (including L1-distance loss, PSNR, and SSIM) and face-feature level (including LightCNN and evoLVe). The NME was also employed to evaluate the proposed method on the task of 3D face reconstruction in comparison with Yang et al. [18], Mobilenetv2 [42], and DeFA [52] on the AFLW2000-3D dataset. Following Ref. [56], the Iterative Closest Points algorithm was first employed to find the corresponding nearest points between the reconstructed 3D face point cloud and ground truth. Then, the NME normalized by the face bounding-box size was calculated. The proposed method showed significant improvements and surpassed the performance of the other three methods on the AFLW2000-3D dataset, as shown in Figure 6. The numerical statistics for each method are listed in Table 3.

#### 4.2.3. Comparisons of Different Network Structures

A complex network structure is generally described as being a deep learning model that often uses forward propagation calculation (required computing power) in addition to calculating its accuracy, combined with the number of parameters (required memory). The proposed method and current mainstream lightweight neural network structures are compared in this section to verify the effectiveness of the proposed network structure on the task of face alignment and in terms of complexity. The experimental network structures included DenseNet [40], MobileNetV2 [42], ResNet50 [57], and the proposed Mobile-FaceRNet. The results of the proposed Mobile-FaceRNet network structure demonstrated a significant reduction in errors on the AFLW and AFLW2000 datasets when compared to the other network models, as listed in Table 4. In terms of operational efficiency, the number of model parameters and Giga-Floating Point Operation (GFLOP) complexity achieved 88.6% and 90.7% reductions, respectively, compared to ResNet50. MobileNetV2 was slightly higher in terms of operational efficiency than Mobile-FaceRNet. However, the proposed method demonstrated an obvious improvement in terms of accuracy. Compared with DenseNet, the number of model parameters and GFLOP complexity achieved 62.3% and 84% reductions, respectively.

For a fairer comparison, the results where Mobile-FaceRNet did not combine the residual attention mechanisms were also calculated. Mobile-FaceRNet was very close to MobileNetV2 in terms of complexity and the number of model parameters while also displaying an obvious accuracy improvement, as shown in Table 4. The proposed method significantly exceeded the performance of the other two network structures in complexity and accuracy on the AFLW and AFLW2000 datasets. In terms of complexity and the number of model parameters, the proposed method achieved 89.5% and 91.6% reductions compared to ResNet50, respectively. Compared with DenseNet, the number of model parameters and GFLOP complexity achieved 65.7% and 86.2% reductions, respectively, showing a significant improvement. In order to better reflect the operating efficiency of our model, we also compared the final computing time of the model. It can be seen that our model is significantly shorter than ResNet50 and DenseNet, and it has the same times as MobileNetv2, but it has significantly improved the detail recovery ability of the model.

#### 4.2.4. Ablation Study

A weakly supervised 3D face reconstruction method was implemented for single image input, and a multiscale feature extraction fusion module and residual attention module were added to the encoder–decoder network. Ablation experiments were performed on the BFM dataset to test the effect of adding a multiscale feature extraction fusion module and a dual attention module to the 3DMM coefficients (s, t, and e), and the experimental results are listed in Table 5 and Table 6, respectively. Two indicators—a scale-invariant depth error (SIDE) and mean angle deviation (MAD)—were used to evaluate the reconstruction effect of the algorithm [56].

SIDE is defined as the error between the reconstructed face depth and the actual face depth, expressed as

(20) $$\mathrm{SIDE}\left(d,\bar{d}\right)=\sqrt{\frac{\sum_{uv}\Delta_{uv}^{2}}{W\times H}-{\left(\frac{\sum_{uv}\Delta_{uv}}{W\times H}\right)}^{2}}$$

where $\bar{d}$ and *d* are the depth values of the reconstructed and actual faces, respectively, and $\Delta_{uv}=\ln\left(d\right)-\ln\left(\bar{d}\right)$.MAD is defined as the average error between the reconstructed face and the surface normal of the actual face, and is expressed as

(21) $$\mathrm{MAD}\left(n,\bar{n}\right)=\frac{\sum_{uv}r\left(n,\bar{n}\right)}{W\times H}$$

where *r* is the angle between the two vectors starting from the same pixel point, *n* is the surface normal vector calculated by using the true depth value of the dataset, and [insert variable here] is the surface normal calculated by using the predicted depth value vector. The ablation results of the two modules are listed in Table 7.

The above results show that adding a single module to the network improved the experimental results to a certain extent, and adding two modules at the same time achieved the best effect. The multiscale feature extraction and fusion module fully combined the semantic information of high-level features with the detailed information of low-level features, strengthened feature transfer and reuse, improved the network gradient disappearance problem, and provided richer feature information for the encoder–decoder prediction network. The residual attention module was added to the encoder and decoder networks to assist the network in better extracting relevant feature information, accelerating the convergence, and completing the reconstruction task.

**Figure 7:**
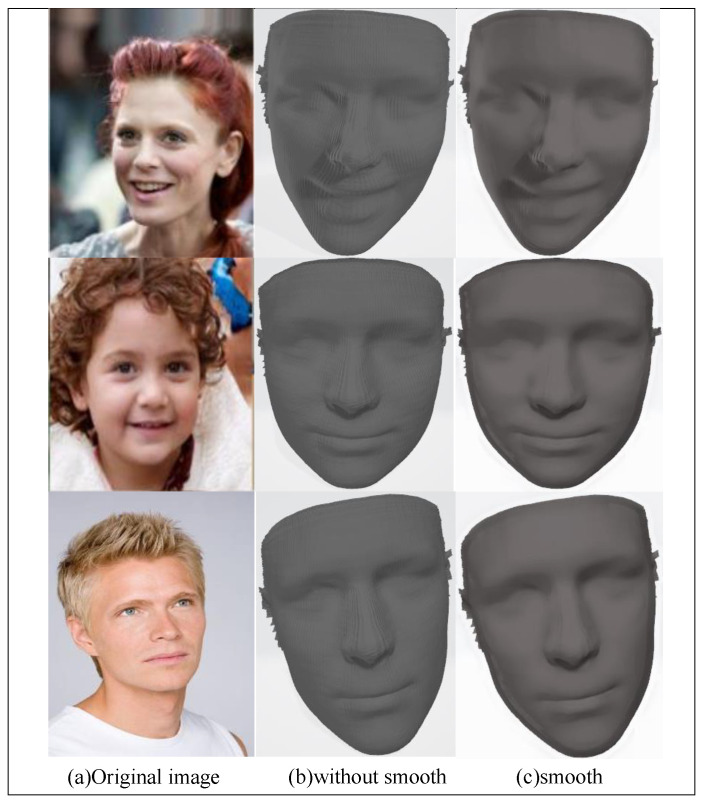
Effect of adding a 3D face smoothness constraint.

An ablation experiment was designed to determine whether to use 3D face smoothness in the loss function, and the effect is shown in Figure 7. Adding the 3D face smoothness constraint to the loss function ensured the local smoothness of the 3D reconstruction model, which had a greater impact on the 3D face reconstruction effect. An image of the input face is shown in Figure 7a. The effects of not using and using the 3D face smoothness constraint are shown in Figure 7b and Figure 7c, respectively. The reconstructed 3D face model had a face flip and a rough surface when the 3D face smoothness constraint was not used, and the quality of the reconstruction was poor. The effect was significantly improved after adding the smoothness constraint. This indicates that the smoothness constraint of the 3D face plays a vital role.

## 5. Conclusions

In this paper, we propose an efficient and lightweight network model, Mobile-FaceRNet, for 3D face reconstruction and dense face alignment improved the ability of the network to extract and process face image features by designing a densely connected multiscale fusion module and introducing a residual attention mechanism. Moreover, the existing 3DMM model was used as part of the fully connected layer of the network. The 3DMM model was improved to effectively reconstruct a more accurate 3D face model and improve its generalization ability. A new loss function was designed that effectively improved the reconstruction quality by adding smoothness constraints to the learned 3D face model and using the SSIM of the input face image and rendered image as the loss. Solves the problem of using a lightweight network for parameter fitting to improve the calculation speed but lose the reconstruction accuracy. Experimental results showed that the proposed method achieved high-precision reconstruction under the premise of a lightweight network; model parameters and GFLOP complexity achieved 65.7% and 86.2% reductions, respectively, showing a significant improvement. At the same time, it was more robust to influences such as attitude and occlusion. This shows that the proposed algorithm has high application value in various scenarios. In future work, we will start with human head reconstruction and consider employing the albedo parameterized model to complement the head texture map and expand the range of reconstruction.

## Figures and Tables

**Table 1:** Mobile-FaceRNet architectural components.

Operator	t	c	n	s
Conv2d	-	32	1	2
Layer1	1	16	1	1
RA Module	-	-	-	-
Layer2	6	24	2	2
RA Module	-	-	-	-
Layer3	6	32	3	2
RA Module	-	-	-	-
Layer4	6	64	4	2
RA Module	-	-	-	-
Layer5	6	96	3	1
RA Module	-	-	-	-
Layer6	6	160	3	2
RA Module	-	-	-	-
Layer7	6	320	1	1
RA Module	-	-	-	-
Conv2d1×1	-	1280	1	1
Avgpool7×7	-	-	1	-
Conv2d1×1	-	k	-	-

**Table 2:** NME (%) results for dense face alignment.

Method	AFLW2000-3D				AFLW-LFPA
	I	II	III	Mean	Mean
SADRNet [49]	-	-	-	4.33	-
Img2pose [50]	-	-	-	3.91	-
3DSTN [51]	3.15	4.33	5.98	4.49	-
DeFA [52]	-	-	-	4.50	3.86
PRNet [34]	3.75	4.51	5.61	5.42	-
Nonlinear 3DMM [16]	-	-	-	4.70	-
ACRLoss [53]	-	-	-	4.27	3.75
Chang et al. [54]	3.11	3.84	6.60	4.52	-
Tran et al. [17]	-	-	-	4.12	-
Ours	2.89	3.76	4.78	3.80	3.34

**Table 3:** Comparative results.

Method	$L_{1}↓$	PNSR↑	SSIM↑	LightCNN↑	evoLVe↑
Deng et al. [35]	0.05	26.58	0.83	0.72	0.64
Gecer et al. [19]	-	26.5	0.898	-	-
Lin et al. [23]	0.034	29.69	0.89	0.90	0.85
Yang et al. [18]	0.02	24.88	0.89	0.91	0.83
Ours	0.01	28.50	0.96	0.94	0.87

**Table 4:** The NME(%) performance comparison of the proposed network against other networks.

Net	AFLW2000-3D			AFLW-LFPA	
	Params (M)	GFLOPs	Time (h)	Mean	Mean
ResNet50 [57]	23.11	1.319	26	4.179	5.471
MobileNetV2 [42]	2.38	0.109	9	4.165	5.540
DenseNet [40]	7.02	0.800	18	4.087	5.286
Ours (no attention)	2.40	0.110	8	3.936	5.201
Ours	2.56	0.121	9	3.828	4.904

**Table 5:** Ablation results of the dual attention module.

Eshape	Etex	Eexp	$\mathrm{SIDE}\left(\times 10^{-2}\right)↓$	$\mathrm{MAD}\left(\deg.\right)↓$
*√*			0.7743	15.8709
	*√*		0.7754	15.8925
		*√*	0.7721	15.7134
*√*	*√*	*√*	0.7637	15.2986

**Table 6:** Ablation results of multiscale feature extraction and fusion module.

Eshape	Etex	Eexp	$\mathrm{SIDE}\left(\times 10^{-2}\right)↓$	$\mathrm{MAD}\left(\deg.\right)↓$
*√*			0.7778	15.6845
	*√*		0.7505	15.1609
		*√*	0.7512	15.4257
*√*	*√*	*√*	0.7160	14.7222

**Table 7:** Ablation results of the two modules.

Eshape	Etex	Eexp	$\mathrm{SIDE}\left(\times 10^{-2}\right)↓$	$\mathrm{MAD}\left(\deg.\right)↓$
*√*			0.7529	15.0565
	*√*		0.7369	15.7608
		*√*	0.7365	15.1116
*√*	*√*	*√*	0.7110	15.4342

## Data Availability

No applicable.
